# Validity of Myocardial Infarction Diagnoses in Administrative Databases: A Systematic Review

**DOI:** 10.1371/journal.pone.0092286

**Published:** 2014-03-28

**Authors:** Natalie McCormick, Diane Lacaille, Vidula Bhole, J. Antonio Avina-Zubieta

**Affiliations:** 1 Faculty of Pharmaceutical Sciences, University of British Columbia, Vancouver, British Columbia, Canada; 2 Arthritis Research Centre of Canada, Richmond, British Columbia, Canada; 3 Division of Rheumatology, Department of Medicine. University of British Columbia, Vancouver, British Columbia, Canada; 4 Co-chair, Cardiovascular Committee of the CANRAD Network, Richmond, British Columbia, Canada; 5 EpiSolutions Consultancy Services, Thane, India; University of Louisville, United States of America

## Abstract

**Background:**

Though administrative databases are increasingly being used for research related to myocardial infarction (MI), the validity of MI diagnoses in these databases has never been synthesized on a large scale.

**Objective:**

To conduct the first systematic review of studies reporting on the validity of diagnostic codes for identifying MI in administrative data.

**Methods:**

MEDLINE and EMBASE were searched (inception to November 2010) for studies: (a) Using administrative data to identify MI; or (b) Evaluating the validity of MI codes in administrative data; and (c) Reporting validation statistics (sensitivity, specificity, positive predictive value (PPV), negative predictive value, or Kappa scores) for MI, or data sufficient for their calculation. Additonal articles were located by handsearch (up to February 2011) of original papers. Data were extracted by two independent reviewers; article quality was assessed using the Quality Assessment of Diagnostic Accuracy Studies tool.

**Results:**

Thirty studies published from 1984–2010 were included; most assessed codes from the International Classification of Diseases (ICD)-9^th^ revision. Sensitivity and specificity of hospitalization data for identifying MI in most [≥50%] studies was ≥86%, and PPV in most studies was ≥93%. The PPV was higher in the more-recent studies, and lower when criteria that do not incorporate cardiac troponin levels (such as the MONICA) were employed as the gold standard. MI as a cause-of-death on death certificates also demonstrated lower accuracy, with maximum PPV of 60% (for definite MI).

**Conclusions:**

Hospitalization data has higher validity and hence can be used to identify MI, but the accuracy of MI as a cause-of-death on death certificates is suboptimal, and more studies are needed on the validity of ICD-10 codes. When using administrative data for research purposes, authors should recognize these factors and avoid using vital statistics data if hospitalization data is not available to confirm deaths from MI.

## Introduction

Cardiovascular diseases (CVD), including myocardial infarction (MI), are associated with physical disability, reduced quality-of-life, economic hardship, and death. In 2008 CVD accounted for 30% of all deaths globally [1], and annual cost estimates for CVD have recently exceeded €169 billion for the European Union [2] and $400 billion in the United States [3]. Although age is one of the primary risk factors for CVD, growing evidence suggests that chronic conditions including inflammatory rheumatic diseases [4]–[9], osteoarthritis [10], diabetes [11], and clinical depression [12] are also associated with an increased risk of CVD, independent of age.

Alongside, there is increasing recognition of the value of administrative data for use in disease surveillance [13]–[19], and this data source has been key in identifying the associations between chronic diseases and CVD as mentioned above. Administrative databases provide easy access to data for a large number of patients attending multiple centres, with longer follow-up periods at relatively low cost. For example, the universal provision of publically-funded health care in Canada allows the patient-level linkage of health resource utilization data (including hospital separations, outpatient visits, procedures and tests, and, in some provinces, dispensed prescriptions) for nearly every resident of each province to demographic and vital statistics data. Consequently, both selection and recall bias are minimized.

Despite these advantages, much uncertainty exists around the validity of diagnoses recorded in administrative data since most databases are not established for research purposes. Instead, records of each healthcare encounter are submitted by physicians and hospital staff primarily to obtain reimbursement. Thus, not all conditions may be recorded in the databases, and those recorded may not correspond to the date of disease onset or reflect the true diagnosis and assessment made by the treating physician. These errors and inconsistencies in diagnostic codes may lead to misclassification bias, impacting the quality of research using these sources and, in turn, any changes in health policy and care practices stemming from it. For example, failure to adequately capture the number of people afflicted by CVD may underestimate the burden of these diseases, thus limiting the health resources allocated to address them. Alternatively, when studying long-term health outcomes, capturing an excess number of false-positive cardiovascular events could overestimate the risks associated with an otherwise beneficial therapy or intervention.

While several assessments of the validity of cardiovascular codes have been published [20]–[23], most concerned a single CVD and were conducted within a limited geographic area, restricting their generalizability. Much inconsistency exists with regards to the methods (including the source of the population and gold standards) adopted by these studies and the way in which results are reported. To our knowledge data on the validity of these codes have not yet been synthesized on a larger scale.

As part of a Canadian Rheumatology Network for establishing best practices in the use of administrative data for health research and surveillance (CANRAD) [13], [19], [24], our objective was to conduct a systematic review of studies reporting on the validity of diagnostic codes for identifying CVD in administrative data. Data from these studies were used to compare the validity of these codes, and to evaluate whether administrative health data can accurately identify CVD for the purpose of identifying these events as covariates, outcomes, or complications in future research. We focus on MI in this paper, and will discuss two other CVD, congestive heart failure and cerebrovascular accident, in subsequent reports.

## Methods

### Literature Search

Comprehensive searches of the MEDLINE and EMBASE databases from inception (1946 and 1974, respectively) to November 2010 for all available peer-reviewed literature were conducted by an experienced librarian (M-DW). Two search strategies were employed: (1) all studies where administrative data was used to identify CVD; (2) all studies reporting on the validity of administrative data for identifying CVD. Our MEDLINE and EMBASE search strategies are available as supplementary materials (Text S1 and S2). To find additional articles, the authors hand-searched the reference lists of the key articles located through the database search. The Cited-By tools in PubMed and Google Scholar were also used to find relevant articles that had cited the articles located through the database search (up to February 2011). The titles and abstracts of each record were screened for relevance by two independent reviewers. No protocol for this systematic review has been published, though the review was conducted in accordance with the Preferred Reporting Items for Systematic Reviews and Meta-Analyses (PRISMA) Statement; our completed PRISMA checklist is provided as supplementary material (Checklist S1). More information about the CANRAD project is available here [13].

### Inclusion Criteria

We selected full-length peer-reviewed articles published in English that used administrative data and reported validation statistics for the International Classification of Diseases (ICD) codes of interest or provided sufficient data enabling us to calculate them. We included studies evaluating particular diagnostic codes for acute MI (being ICD-8 & ICD-9 code 410 and ICD-10 codes I21&I22) and excluded studies that evaluated umbrella diagnoses. This means we did not include validity statistics from studies where other codes were included in the algorithm for MI (ie. 410–411 or 410–414). For example, the MI statistics in one study [25] were not included because the algorithm included a code for cardiac arrest (ICD-9 427.5); those in three others [26]–[28] were not included because those algorithms contained codes for old MI (ICD-9 412 and ICD-10 code I25.2). Any discrepancies were discussed until consensus was reached. When the conflict persisted a third reviewer (JAA-Z) was consulted.

### Data Extraction

The full text of each selected record was examined by two independent reviewers (NM and VB) who abstracted data using a standardized collection form (a copy is provided in Text S3) developed for the CANRAD investigations. While extracting data, particular attention was given to the study population, administrative data source, algorithm used to identify the CVDs, validation method and gold standard. Validation statistics comparing the MI codes listed above to definite, probable, or possible cases were abstracted. These statistics included sensitivity, specificity, positive predictive value (PPV), negative predictive value (NPV), and kappa scores. Because hospital separations typically contain multiple diagnoses, with the primary or principle diagnosis in the first position followed by one or more secondary diagnoses, we abstracted statistics for each of these positions, where available. Data were independently abstracted by each reviewer, who subsequently compared their forms to correct any errors and resolve discrepancies.

The design and methods used by each study (for example, whether or not the diagnosis recorded in the administrative database formed part of the reference standard) can directly influence the validity statistics produced. Thus, all studies were evaluated for quality, and the validation statistics were stratified by level of study quality. We used the Quality Assessment of Diagnostic Accuracy Studies (QUADAS) tool [29] (available as a part of Text S3), used previously by the CANRAD network in assessing the validity of codes for osteoporosis and fractures [30]. Briefly, it is a 14-item evidence-based quality assessment tool used in systematic reviews of diagnostic accuracy studies. Each item, phrased as a question, addresses one or more aspects of bias or applicability; however, there is no overall score. Instead, as done previously [30], items were independently answered by each reviewer and used to qualitatively assess each study as High, Medium, or Low quality. Any disagremeents were resolved by consensus.

### Statistical Analysis

All validation statistics were abstracted as reported. Where sufficient data were available we calculated 95% confidence intervals (95% CI) and additional validity statistics not directly reported in the original publication. For each CVD these were evaluated on aggregate, and, as pre-specified, stratified by administrative data source (ie. hospitalization vs. vital statistics). Sensitivity (the ability of the codes to identify true positive cases) was equal to the number of true positives divided by the sum of true positives and false negatives (all those who are diseased). Specificity (the ability of the codes to exclude false-positive cases) was equal to the number of true negatives divided by the sum of true negatives and false positives (all those who are non-diseased). PPV (the likelihood that the code corresponds to a true-positive case) was equal to the number of true positives divided by the total number of cases receiving the code (true-positives and false-positives). NPV (the likelihood that a record not coded for the condition is a true-negative case) was equal to the number of true negatives divided by the total number of cases without the code (true-negatives and false-negatives). Kappa (a measure of agreement beyond that expected by chance) is equal to the observed agreement minus that expected by chance, divided by [100% - the agreement expected by chance]. Values greater than 0.60 indicate substantial/perfect agreement, 0.21–0.60 were considered as fair/moderate agreement and those 0.20 or lower as light/poor agreement [31].

Where available, we abstracted statistics for definite, probable, and possible cases of MI. However the choice of gold standard dictates the number of categories reported, and some studies will classify cases simply as MI or no MI. Under the American Heart Association (AHA) [32] and Joint European Society of Cardiology/American College of Cardiology (ESC/ACC) criteria, true-positive cases are classified as either definite, probable, possible, or no MI. However, the MONICA criteria, used in the World Health Organization (WHO) 's Multinational MONItoring Trends and Determinants in CArdiovascular Disease project, only uses three categories. Briefly, the MONICA project was conducted over 10 years (during the 1980's and 1990's) across 32 study areas in 21 countries to monitor trends in cardiovascular diseases and changes in risk factors [33]. As part of the study, all suspected coronary events in those aged 25–64 years were entered into a registry. Suspected events were identified prospectively (while cases were in hospital) and retrospectively (by examining hospital databases and death certificates), and study physicians used the MONICA criteria to classify these events as definite, possible or no MI [33]. The criteria considered symptoms, electrocardiogram (EKG) findings and cardiac enzyme levels when making the diagnosis. ‘Definite’ cases are the most certain because they meet the strictest criteria for each CVD (enzyme levels and EKG in addition to typical symptoms) while ‘Possible’ cases include typical symptoms only [33]. Because more potential cases are expected to fulfil the broader criteria for ‘Definite or Possible’, the PPV for this broader category should be greater. However, this comes at a cost to specificity since more false-positives will meet these broader criteria too.

## Results

### Literature Search

**Figure 1:**
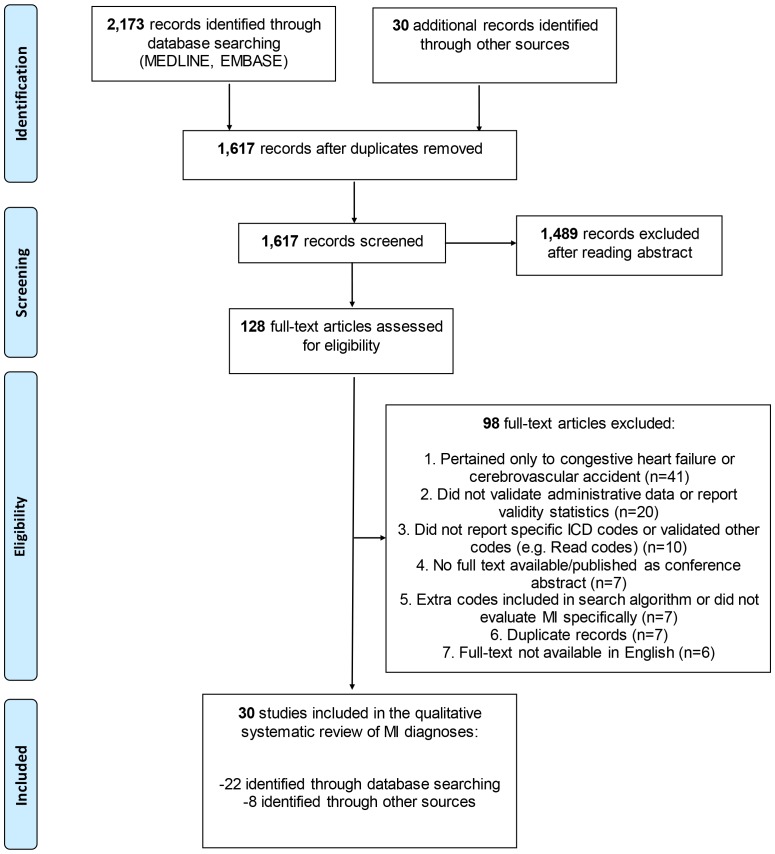
Preferred Reporting Items for Systematic Reviews and Meta-Analyses (PRISMA)-style Flowchart of Study Selection and Review. ICD = International Classification of Diseases; MI = myocardial infarction.

After the removal of duplicates, 1,587 citations were identified through MEDLINE and EMBASE searches and screened for relevance to our study objectives. We then assessed 98 full-text articles for eligibility (**Figure 1**), of which 22 were selected for inclusion. We also assessed 30 full-text articles for eligibility that were identified from other sources, and selected 8 additional articles therein. This meant a total of 128 articles were assessed for eligibility, from which 98 were excluded, mainly because they reported on the validity of other CVD (n = 41), or did not actually validate MI diagnoses in administrative data (n = 20). Six articles were excluded because they were not published in English; their languages of publication were Danish, German, Italian, Japanese, Portugese, and Spanish. Ultimately, 30 articles were included for the systematic review of MI.

### Study Characteristics

Of the 30 studies evaluating MI diagnoses that were included in the final review, 12 (40%) were from Europe, 8 (27%) were from the United States (USA), 7 (23%) were from Canada, 2 (7%) were from New Zealand, and 1 (3%) was from Australia. Characteristics of these studies are presented in **Table 1**. Validation was the primary research objective in 26 (87%) of them. Altogether data were collected over a 34-year period (1970 to 2003) that covered three revisions of the ICD system (ICD-8, ICD-9, and ICD-10). Nearly all administrative data sources pertained to hospitalizations with algorithms consisting of ICD diagnostic codes but no procedure codes. Five studies evaluated the validity of MI as a cause-of-death on death certificates, but none of the studies evaluated diagnoses for outpatient encounters. National and regional disease registries and surveillance systems served as the gold standards in 10 (33%) studies [20], [21], [34]–[41]. In the 20 remaining studies, the gold standards were based on chart reviews, often in consultation with established diagnostic criteria. Just two studies [42], [43] reported on the validity of ICD-10 codes separately from ICD-9 codes.

**Table 1:** Characteristics of included studies.

First Author, Year of Publication	Year(s) of Data Collection	Primary Validation Study?	Country	Records Evaluated (N)	Source Population	Type of Administrative Data	Gold Standard	Cardiac Troponin Levels Included in the Reference Standard?
**Ainla** [42], 2006	2001	no	Estonia	520	general hospitalized population	ICD-10 inpatient records	CRDC - Joint European Society of Cardiology/American College of Cardiology Committee criteria	yes
**Austin** [48], 2002	1996–2000	yes	Canada (Ontario)	20,048	consecutive patients 20 years+ admitted to in coronary care units from ER (excluded patients transferred from another institution, with urgent or emergent admission, if most responsible diagnosis didn't occur during hospital stay)	ICD-9 inpatient records	disease registry - diagnosis of bedside nurse (as recorded in Fastrak II prospective acute coronary syndromes registry)	n/a
**Barchielli** [49], 2010	2003	yes	Italy	372	from population-based Tosc-AMI registry in Tuscany, hospitalized patients, all out-of-hospital deaths	ICD-9 inpatient records	CRDC - American Heart Association (AHA) and MONICA criteria	AHA = yes, MONICA = no
**Beaglehole** [37], 1987	1983	yes	New Zealand	604	general hospitalized population from 3 general hospitals (aged 25–64)	ICD-9 inpatient records	disease registry - MONICA project	no
**Boyle** [20], 1995	1986–1991	yes	Australia	2,492	all residents of 5 communities aged 25–69 years	ICD-9 inpatient records; death certificates	disease registry - MONICA project	no
**De Henauw** [39], 1998	1983–1991	yes	Belgium	1,675	general population of two Belgian cities	ICD-9 death certificates	disease registry - MONICA project	no
**Hammar** [64], 2001	1987, 1995	no	Sweden	1,848	all hospitalized patients and all deaths in the country	ICD-9 inpatient records; death certificates	CRMD	yes
**Heckbert** [65], 2004	1994–2000	yes	USA (national)	807	women participating in the Women's Health Initiative clinical and observational studies	ICD-9 inpatient records	CRDC - Women's Health Initiative criteria	yes
**Jackson** [38], 1988	1983–1984	yes	New Zealand	411	general population <65 years residing in Auckland	ICD-9 death certificates	disease registry - MONICA project	no
**Kennedy** [40], 1984	not provided	yes	USA (Texas)	110	hospitalized patients at large urban teaching hospital	ICD-9 inpatient records	disease registry - Cardiology Surveillence System (cardiologyservice)	n/a
**Kiyota** [47], 2004	1999–2000	yes	USA (Pennsylvania)	2,022	hospitalized elderly Medicare beneficiaries in one US state	ICD-9 inpatient records	CRDC – modified WHO criteria	yes
**Levy** [45], 1999	1994	yes	Canada	234	individuals ≥65 years hospitalized with MI (primary diagnosis)	ICD-9 inpatient records	chart review: mentioned in records	n/a (no formal diagnostic criteria)
**Lindblad** [66], 1993	1977–1987	yes	Sweden	413	participants in hypertension registry (hypertensive cases, normotensive participants, randomly-selected controls), recruited from a geographical half of one Swedish county, aged 40–70 years at registration	ICD-9 inpatient records	CRMD	no
**Lowel** [41], 1991	1985–1986	yes	Germany	753	hospitalized patients aged 25–74	ICD-9 inpatient records, death certificates	disease registry - MONICA project	no
**Mahonen** [35], 1997	1983–1990	yes	Finland	4,836	from general population 25–64 years, all hospitalized patients residing in FINMONICA study areas; also screened hospitalized stroke cases to calculate sensitivity for MI	ICD 8,9 inpatient records	disease registry - FINMONICA project	no
**Mahonen** [34], 1999	1983–1992	yes	Finland	3,182	general population aged 35–64 years residing in FINMONICA study areas	ICD-8,9 death certificates	disease registry - MONICA project	no
**McCarthy** [44], 2000	1994	yes	USA (California, Connecticut)	485	hospitalized Medicare beneficiaries 65 years+	ICD-9 inpatient records	CRMD	unknown
**Merry** [62], 2009	1987–2003	yes	Netherlands	417	residents of one regions in the Netherlands born between 1927–1977 (aged 20–59 years at registration) participating in two large monitoring projects	ICD-9 inpatient records	disease registry - diagnosis listed in Cardiology Information System (hospital cardiology service)	n/a
**Nova Scotia-Saskatchewan Cardiovascular Disease Epidemiology Group** [51],1989	1974–1977	no	Canada (Nova Scotia & Saskatchewan)	813	general hospitalized population (from all residents aged 25–74)	ICD-8 inpatient records	CRDC - MONICA criteria	no
**Nova Scotia-Saskatchewan Cardiovascular Disease Epidemiology Group** [50],1992	1977, 1981, 1985	no	Canada (Nova Scotia & Saskatchewan)	1,810	general hospitalized population (from all residents aged 25–74)	ICD inpatient records	CRDC - MONICA criteria	no
**Pajunen** [43], 2005	1988–2002	yes	Finland	37,062	general population (all hospitalizations and deaths) residing in FINMONICA study areas	ICD-9 and ICD-10 inpatient records; death certificates	chart review: using American Heart Association criteria	yes
**Palomaki** [21], 1994	1987–1990	yes	Finland	1,565	people aged 35–64 years hospitalized for suspected CHD	ICD-9 inpatient records	disease registry - FINMONICA and MONICA project	no
**Petersen** [67], 1999	1994–1995	yes	USA	4,712	hospitalized male veterans	ICD-9 inpatient records	CRDC - Cooperative Cardiovascular Project criteria	no
**Pladevall** [22], 1996	1988–1990	yes	USA (Texas)	734	hospitalized participants in Corpus Christi Heart Project (MONICA-affiliated surveillance program in one Texas county) aged 25–74 years, Mexican-American and non-Hispanic white	ICD-9 inpatient records	CRDC - Cardiovascular Community Surveillence Project criteria	no
**Rapola** [46], 1997	1985–1993	yes	Finland	408	male smokers aged 50–69 years at registration participating in cancer prevention study	ICD-8,9 inpatient records; death certificates/death register	CRDC - FINMONICA critera	no
**Rawson** [68], 1995	1986	yes	Canada (Saskatchewan)	224	general hospitalized population	ICD-9 inpatient records	CRMD	unknown
**Rosamond** [36], 2004	1987–2000	yes	USA (North Carolina, Mississippi, Minnesota, Maryland)	17,900	from general population (participants in population-based ARIC study), all hospitalized patients aged 35–74, excluded non-whites or blacks	ICD-9 inpatient records	disease registry - Atherosclerosis Risk in Communities project	yes, 1996-onwards
**van Walraven** [69], 1990	1987–1988	yes	Canada (Ontario)	209	hospitalized patients at tertiary hospital	ICD-9 inpatient records	CRMD	no
**Varas-Lorenzo** [70], 2008	1999–2001	yes	Canada (Saskatchewan)	193	as part of a population-based cohort study, hospitalized patients aged 40–84 years	ICD-9 inpatient records	CRDC - American Heart Association/European Society of Cardiology criteria	yes
**Wahl** [71], 2010	2002–2004	yes	USA	200	commercially-insured individuals in large health claims database	ICD-9 inpatient records	CRMD	unknown

CRDC = Chart Review, Diagnostic Criteria – the charts of potential cases were reviewed, and a formal set of diagnostic criteria were applied when evaluated cases.

CRMD = Chart Review, Medical Doctor – the charts of potential cases were reviewed by a physician, who evaluated cases using their clinical judgement or an otherwise unspecified set of criteria.

AHA = American Heart Association; MONICA =  MONItoring Trends and Determinants in CArdiovascular Disease; WHO = World Health Organization.

Study quality was evaluated based on the QUADAS tool [29], with 26 of 30 studies (87%) categorized as high quality, and four (13%) as medium quality. A detailed breakdown of the evaluations for each study is provided in Table S1. In one of the medium-quality studies [44] the validation process was not adequately described, while the gold standard in another [45] was considered less-reliable because charts of potential MI cases were not evaluated by a clinician. The two other medium-quality studies employed a select source population – male smokers aged 50–69 years in one [46], and those aged 65 years or older in another [47] - which limited their generalizability.

PPV data were available from all but one study [39] while the kappa statistic was reported in only two studies [21], [48]. Sensitivity, specificity, and NPV were less-frequently reported by authors, but sufficient data to allow calculation of these statistics were often available and included when the source population was sufficiently broad (ie. when it was not confined to cases receiving codes of ICD-9 410–414, which correspond to a more general category of coronary heart diseases that includes MI).

### Validity of Myocardial Infarction Diagnoses

The validation statistics reported by each of the included studies are provided in **Table 2**. Sensitivity was reported by 12 studies, and was at least 86% in half of them. PPV, obtained from 29 studies, was ≥93% in the majority (n = 15) of them. Specificity and NPV were available only from three studies [22], [40], [48] and in these ranged from 89–99%, and 75–99%, respectively. Five studies [34]–[36], [43], [45] provided sex-stratified statistics and in four of these [35], [36], [43], [45] sensitivity and PPV values were higher for males (**Table 2**). Twenty-six of the 30 studies on MI (87%) were of high quality and the PPV was ≥80% in 20 of 25 (80% of the high-quality studies). One high-quality study [39] did not report PPV. One of the medium-quality studies reported a PPV of 81% [44], while in the three others [45]–[47] this value ranged from 95–98%. None of the medium-quality studies reported on sensitivity, specificity, NPV, or kappa.

**Table 2:** Results of studies validating diagnoses of myocardial infarction (MI) in administrative data (in ascending order of publication).

First Author, Year	Diagnostic Codes	Parameter	Sensitivity (95% CI)	Specificity (95% CI)	PPV (95% CI)	NPV (95% CI)	Kappa (95% CI)	Quality	
**Kennedy**, 1984 [40]	ICD9 410		94.37 (85.46–98.18)	99.79 (99.71–99.85)	60.91 (51.11–69.93)	99.98 (99.95–99.99)		High	
**Beaglehole**, 1987 [37]	ICD9 410	definite MI	85.99 (82.45–88.93)		67.05 (63.12–70.76)			High	
**Jackson**, 1988 [38]	ICD9 410	definite MI	84.03 (78.61–88.32)		48.66 (43.74–53.60)			High	
**Nova Scotia-Saskatchewan Cardiovascular Disease Epidemiology Group**, 1989 [51]	ICD8 410 or ICD9 410 (primary or secondary hospital diagnosis, or underlying COD)	definite MI, overall			57.56 (54.08–60.98)			High	
		definite or possible MI, overall			80.44 (77.57–83.02)				
**van Walraven**, 1990 [69]	ICD9 410	primary discharge diagnosis			79.43 (73.18–84.56)			High	
**Lowel**, 1991 [41]	ICD9 410	nonfatal definite MI			73.44 (70.10–76.53)			High	
		nonfatal definite or possible, MI			89.38 (86.90–91.44)				
**Nova Scotia-Saskatchewan Cardiovascular Disease Epidemiology Group**, 1992 [50]	ICD 410	definite or possible MI			85.47 (83.74–87.04)			High	
**Lindblad**, 1993 [66]	ICD8 410.00 or 410.99; or ICD9 410A-X	definite MI			95.64 (93.07–97.32)			High	
		definite or possible MI			98.79 (97.03–99.55)				
**Palomaki**, 1994 [21]	ICD9 410.0	definite MI, vs. MONICA			86.40 (83.23–89.06)		0.81 (0.76–0.86)	High	
	410.0 or 410.9	definite MI, vs. MONICA			70.98 (67.53–74.21)				
	410.0 or 410.9	definite or possible MI, vs. MONICA			93.46 (91.36–95.09)		0.25 (0.21–0.29)		
	410.0	definite MI, vs. FINMONICA			86.4 (83.23–89.06)		0.81 (0.76–0.86)		
	410.0 or 410.9	definite MI, vs. FINMONICA			70.98 (67.53–74.21)				
	410.0 or 410.9	definite or possible MI, vs. FINMONICA			91.96 (89.69–93.78)		0.60 (0.55–0.65)		
	410.0	definite or possible MI, vs. MONICA classification			96.47 (94.50–97.77)				
		definite or possible MI, vs. FINMONICA			95.94 (93.87–97.35)				
**Rawson**, 1995 [68]	ICD9 410	primary discharge diagnosis			96.88 (93.40–98.62)			High	
**Boyle**, 1995 [20]	ICD9 410	definite MI (hospital)	78.90 (77.10–80.77)		65.57 (63.66 – 67.43)			High	
		definite or possible MI (hospital)	43.18 (41.77–44.61)		81.74 (80.16–83.23)				
		definite MI(death certificate)	79.85 (75.49–83.62)		25.56 (23.18–28.11)				
		definite or possible MI (death certificate)	72.66 (70.09–75.09)		73.71 (71.15–76.12)				
**Pladevall**, 1996 [22]	ICD9 410	definite MI	80.9 (77.4–84.4)	93.1 (92.4–93.8)	54.6 (53.2–56.0)	97.9 (96.7–99.2)		High	
		definite or possible MI	36.3 (34.1–38.5)	97.5 (97.0–98.0)	87.7 (86.6–88.8)	75.4 (73.4–77.4)			
**Mahonen**, 1997 [35]	ICD8 410	definite MI: men	84.4 (83.2–86.3)		79.5 (77.8–81.1)			High	
		definite or possible MI: men	72.0 (74.3–79.7)		90.7 (89.6–91.8)				
		definite MI: women	85.5 (82.3–88.7)		72.5 (68.8–76.2)				
		definite or possible MI: women	62.2 (59.2–65.3)		87.5 (85.0–90.0)				
	ICD9 410	definite MI: men	86.0 (84.4–87.6)		85.9 (84.2–87.5)				
		definite or possible MI: men	66.8 (65.1–68.5)		93.3 (92.3–94.4)				
		definite MI: women	81.3 (77.6–85.0)		80.7 (77.0–84.4)				
		definite or possible MI: women	55.9 (52.6–59.2)		89.6 (87.1–92.2)				
**Rapola**, 1997 [46]	ICD8 410 or ICD9 410	definite nonfatal MI			77.88 (71.65–83.10)			Medium	
		definite or possible nonfatal MI			93.55 (89.19–96.30)				
		definite fatal MI			45.31 (36.58–54.33)				
		definite or possible fatal MI			95.31 (89.64–98.08)				
		definite fatal & nonfatal MI			65.80 (60.49–70.74)				
		definite or possible fatal & nonfatal MI			94.20 (91.05–96.33)				
**De Henauw**, 1998 [39]	ICD9 410	definite or possible MI, as underlying cause of death	49.13 (46.72–51.56)					High	
**Mahonen**, 1999 [34]	ICD8 410	definite MI, men	91.34 (89.04–93.21)		54.27 (51.48–57.04)			High	
		definite MI, women	89.81 (83.71–93.88)		58.51 (51.99–64.75)				
		definite MI, overall	91.08 (88.99–92.81)		54.95 (52.39–57.48)				
	ICD9 410	definite MI, men	89.14 (86.85–91.09)		55.01 (52.37–57.61)				
		definite MI, women	90.00 (84.23–93.89)		59.07 (52.80–65.07)				
		definite MI, overall	89.28 (87.21–91.06)		55.64 (53.22–58.03)				
	ICD8 410	definite or possible MI, men	71.96 (69.76–74.07)		97.07 (95.95–97.90)				
		definite or possible MI women	74.59 (69.27–79.29)		95.02 (91.25–97.28)				
		definite or possible MI, overall	72.37 (70.35–74.30)		96.74 (95.68–97.56)				
	ICD9 410	definite or possible MI, men	66.96 (64.88–68.98)		97.6 (96.63–98.31)				
		definite or possible MI, women	69.04 (63.98–73.70)		97.3 (94.28–98.81)				
		definite or possible MI, overall	67.27 (65.36–69.13)		97.56 (96.67–98.22)				
**Levy**, 1999 [45]	ICD9 410, as primary discharge diagnosis	overall			95.73 (93–98)			Medium	
		men			97.74 (93.05–99.42)				
		women			93.07 (85.76–96.92)				
**Petersen**, 1999 [67]	ICD9 410	primary discharge diagnosis			96.88 (96.33–97.35)			High	
**McCarthy**, 2000 [44]	ICD9 410	compare w/objective clinical evidence			81.08 (64.29–91.45)			Medium	
**Hammar**, 2001 [64]	ICD9 410	definite MI			85.83 (83.01–88.27)			High	
		definite or possible MI			94.95 (93.01–96.39)				
**Austin**, 2002^a^ [48]	ICD9 410	primary position	88.83 (88.41–89.24)	92.84 (92.57–93.11)	88.54 (88.12–88.95)	93.03 (92.76–93.29)	0.82	High	
		primary or secondary position	92.8	89.2	84.2				
**Rosamond**, 2004 [36]	ICD9 410	definite MI, overall	67.99 (67.23–68.75)		55.98 (55.25–56.71)			High	
		definite or probable MI, overall	63.26 (62.61–63.91)		75.26 (74.62–75.89)				
		definite MI, men	69		58				
		definite or probable MI, men	65 (63–66)		77				
		definite MI, women	66		52				
		women definite or probable MI, women	60 (58–62)		73				
**Heckbert**, 2004 [65]	ICD9 410 only	MI			83.02 (80.21–85.51)			High	
**Kiyota**, 2004^b^ [47]	ICD9 410.01, 410.11, 410.21, 410.31, 410.41, 410.51, 410.61, 410.71, 410.81, 410.91	primary or secondary admission position, 3≤LOS≤180 days			94.11 (92.92–95.12)			Medium	
		primary admission position, 3≤LOS≤180 days			95.1 (94.1–96.2)				
	ICD9 410.X0, 410.X1, and 410.X2	primary or secondary admission position, 3≤LOS≤180 days			92.45 (91.13–93.59)				
**Pajunen**, 2005^c^ [43]	ICD9 410 or ICD10 I21-I22	definite/probable/possible nonfatal MI	61–81		79–93			High	
**Ainla**, 2006 [42]	ICD10 I21-I22	tertiary care hospitals			93.3			High	
		secondary care hospitals			83.5				
**Varas-Lorenzo**, 2008 [70]	ICD9 410	definite MI			72.02 (65.03–78.11)			High	
		definite or probable/possible MI			94.82 (90.40–97.35)				
**Merry**, 2009 [62]	ICD9 410 for hospital; ICD9 410 or ICD10 I21-I22 as COD	MI overall	83.99 (80.34–87.09)		96.88 (94.59–98.26)			High	
**Wahl**, 2010 [71]	ICD9 410.xx (excluding 410.x2)	true or probable MI overall			88.50 (83.05–92.42)			High	
**Barchielli**, 2010 [49]	ICD9 410 as primary discharge diagnosis	definite MI, vs. AHA criteria			86.02 (81.98–89.30)			High	
		definite or probable MI, vs. AHA criteria			87.37 (83.46–90.48)				
		definite or probable or possible MI, vs. AHA criteria			94.62 (91.68–96.60)				
		definite MI, vs. MONICA criteria			52.69 (47.48–57.84)				
		definite or probable MI, vs. MONICA criteria			65.32 (60.21–70.11)				

a Validation was conducted amongst a coronary care unit population.

b Eight algorithms were evaluated in this study; the major parameters are listed here.

c This article only reported statistics specific to each sex, age category, and time period, and did not report on the validity of nonfatal MI overall; therefore, we have provided the ranges of the 10 sensitivity and PPV values reported in this article.

COD = cause-of-death; LOS = length-of-stay; 95% CI = 95% confidence interval.

In order to examine secular trends in the validity of MI codes, the studies in Tables 2 and 3 have been ordered chronologically by publication year. Half of the MI studies were published between 1984 and 1998, and the other half from 1999 to 2010. No clear trends in sensitivity were observed amongst the twelve studies reporting this statistic. However, at least amongst studies providing statistics on hospitalization data, we did observe somewhat of a trend towards higher PPV's in later years: the PPV was ≥89% in eight of the ten most-recent studies (from 2002 to 2010) while only four out of the 10 earliest studies (from 1984 to 1995) reported PPV≥89%. Of interest, Rosamond *et al* [36] analysed the validity of MI diagnoses recorded from 1987 to 2000, with no secular trends overall in sensitivity or PPV. We were unable to directly evaluate any secular trends in specificity or NPV as there were very few studies (n = 3) reporting these statistics.

**Table 3:** Results of studies validating diagnoses of myocardial infarction (MI) as a cause-of-death (COD) in vital statistics data (in ascending order of publication).

First Author, Year	Diagnostic Codes	Parameter	Sensitivity (95% CI)	PPV (95% CI)	Quality
**Jackson**, 1988 [38]	ICD9 410	definite MI	84.03 (78.61–88.32)	48.66 (43.74–53.60)	High
**Boyle**, 1995 [20]	ICD9 410	definite MI	79.85 (75.49–83.62)	25.56 (23.18–28.11)	High
		definite or possible MI	72.66 (70.09–75.09)	73.71 (71.15–76.12)	
**Rapola**, 1997 [46]	ICD8 and ICD9 410	definite MI		45.31 (36.58–54.33)	Medium
		definite or possible MI		95.31 (89.64–98.08)	
**De Henauw**, 1998 [39]	ICD9 410	definite or possible as underlying COD	49.13 (46.72–51.56)		High
**Mahonen**, 1999 [34]	ICD8 410	definite MI	91.08 (88.99–92.81)	54.95 (52.39–57.48)	High
		definite or possible MI	72.37 (70.35–74.30)	96.74 (95.68–97.56)	
	ICD9 410	definite MI	89.28 (87.21–91.06)	55.64 (53.22–58.03)	
		definite or possible MI	67.27 (65.36–69.13)	97.56 (96.67–98.22)	

COD = cause-of-death.

As expected, there was also some variability in results with regards to the selection of gold standard and specific diagnostic criteria. The MONICA criteria, described above, were used in 12 studies [20], [21], [34], [35], [37]–[39], [41], [46], [49]–[51], and the sensitivity and PPV in these was lower than in studies using the current criteria. For example, the reported sensitivity of ICD 410 for detecting cases of definite or possible MI using the MONICA was 43% [20] in one study and ranged from 56–72% [34] in another. However, the PPV was noticeably higher (94–95% in the primary or secondary admission position) [47] in one article where levels of an additional biomarker of cardiac damage, troponin, were considered in addition to the standard MONICA criteria. In one study comparing the PPV's associated with two gold standards, the PPV for definite MI was 86% using American Heart Association (AHA) criteria but only 53% using MONICA criteria [49]. Finally, while it wasn't consistent across all studies using the MONICA criteria, the PPV's were generally higher in those that were part of an actual MONICA registry [20], [21], [34], [35], [37], [38], [41] than in other investigations that simply used the MONICA criteria to evaluate potential cases of MI [46], [49]–[51].

**Figure 2:**
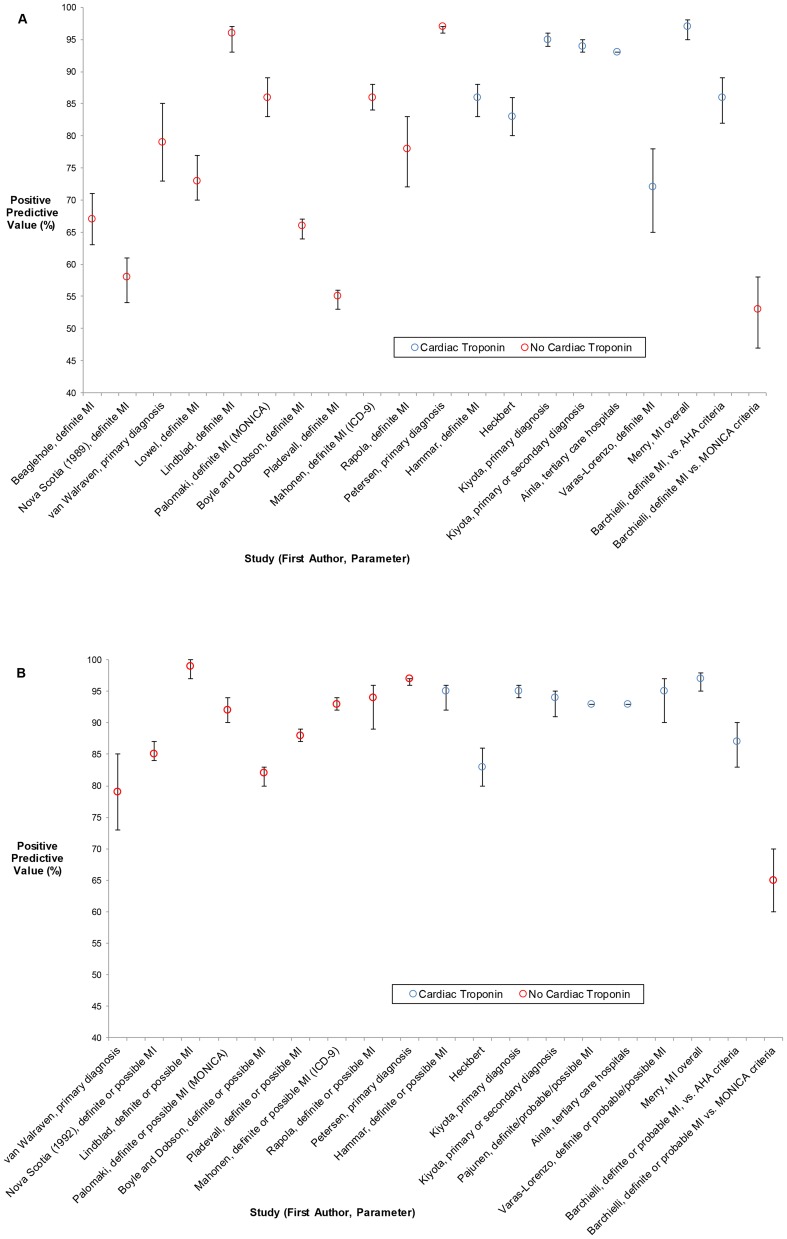
Positive Predictive Values of Myocardial Infarction Diagnoses (versus “Definite” or “Definite/Probable/Possible MI”, or parameters unspecified). The positive predictive values (PPV's) and 95% confidence intervals (where reported) from studies that validated myocardial infarction (MI) diagnoses in hospitalization data, and included a formal set of diagnostic criteria in the reference standard, are ordered left-to-right by publication year of the study (with the earliest-published study on the far left). The PPV's are also stratified by whether cardiac troponin testing was incorporated in the diagnostic criteria. Illustrated in Panel A are the PPV's calculated when the coded diagnoses were compared to the stricter parameter of “Definite MI”, and the PPV's for which no parameter was specified. Illustrated in Panel B are the PPV's calculated when the coded diagnoses were compared to the broader parameter of “Definite and Probable or Possible MI”, along with the same PPV's in Panel A for which no parameter was specified.

The PPV values from studies that reported on hospitalization data and incorporated a formal set of diagnostic criteria in their gold standard are plotted in **Figure 2**. The studies are ordered chronologically by year of publication. **Figure 2a** contains the estimates pertaining to the stricter parameter of “Definite MI”, and **Figure 2b** contains the estimates pertaining to the broader parameter of “Definite or Probable or Possible MI”, as estimates for these two parameters cannot be directly compared. If no parameter was specified in the study (ie. the MI code was compared to a diagnosis of simply “myocardial infarction”), we include that estimate in both figures. To allow for visual inspection of the impact of cardiac troponin measurement on the PPV of MI diagnoses, the PPV's in **Figure 2** are colour-coded as to whether or not levels of cardiac troponin were included in the diagnostic criteria.

We also stratified results by geographic regions (Europe, the South Pacific (Australia and New Zealand), Canada, and the USA), and there was little difference in the sensitivity values reported in each region (**Table 2**). Similarly, there were few differences in the PPV's from different regions; this value was >80% in most of the Canadian and US studies, and ≥89% in all 11 European studies reporting this statistic. However, the PPV's in the three studies from the South Pacific were comparatively lower, with values ranging from 49 [38] –82% [20].

In most studies [≥50%] providing hospital statistics, PPV values were ≥93%, but the accuracy of MI as a cause-of-death on death certificates was much lower. For example, the PPV for definite MI amongst these studies was <60% (**Table 3**), while in many of the studies from hospitalization databases the PPV for definite MI was ≥86% when using the strictest category.

## Discussion

To our knowledge this is the first systematic review on the validity of MI diagnoses in administrative data. Overall, MI diagnostic codes from hospitalization data appear to be valid: in more than half of the studies, sensitivity and specificity exceeded 83%, and PPV exceeded 92%. Therefore, we believe hospitalization data can be used to identify MI either as a covariate or as an outcome. The accuracy of MI as a cause of death on death certificates was lower, with the highest PPV for definite fatal MI being 59% amongst the studies included. In comparison, the PPV was greater than 59% in three-quarters of the studies reporting on hospitalization data. Accordingly, caution should be taken when using vital statistics data to identify deaths from MI, and authors are encouraged to acknowledge this limitation.

It is possible that our findings on the accuracy of MI diagnoses were unduly influenced by publication bias or selective outcome reporting, wherein some authors who did assess the validity of MI codes in their study may have chosen not to report the statistics if they were low. But while our findings for MI in hospitalization data were generally positive, there were exceptions. For example, we observed that the accuracy of MI diagnoses was heavily influenced by the gold standard employed, with lower statistics when the previously-used, more conservative MONICA criteria [52] were applied. These criteria, developed in the 1970's and 80's from international standards, differ from more recent criteria with regards to the biomarkers of cardiac damage. The creatine kinase, lactate dehydrogenase, and aspartate transaminase enzymes are part of MONICA [33], used by 12 studies in this review [20], [21], [34], [35], [37]–[39], [41], [46], [49]–[51]. Three studies [43], [49], [53] used the 2003 American Heart Association (AHA) criteria, which consider levels of cardiac troponin [32] - a component of cardiac muscle and a more sensitive and specific indicator of myocardial damage [54] – in addition to creatine kinase. Similarly, in the Joint European Society of Cardiology/American College of Cardiology (ESC/ACC) criteria [55] - used in two studies [42], [53] - troponin levels take precedence over creatine kinase, and neither aspartate transaminase nor lactate dehydrogenase (the two other enzymes from MONICA) are considered markers of cardiac damage [56].

Support for the increased sensitivity of cardiac troponin is provided by many clinical and population-based studies [57]–[59] where more cases of MI were detected when applying the new criteria than when the MONICA. Consistent with this, some authors have shown that, when defined by the older criteria, the incidence of MI appears to have declined over the decades, but when the newer criteria are applied, the incidence appears to have remained steady [60] or even increased [61]. In other words, more cases will be classified as MI under the newer criteria than the old. Thus, given the increased sensitivity of the newer criteria, we expected to see greater sensitivity values amongst the more recently-published studies in this review, but we did not observe a trend in either direction. Amongst the ten studies reporting on the sensitivity of MI diagnoses in hospital data, sensitivity in the five earlier studies ranged from 80–94%, while in the five later studies it ranged similarly from 69–93%. This may simply be due to the comparatively small number of studies where sensitivity was reported, though heterogeneity in the study settings may also play a role. One study included in our review, by Rosamond *et al* [36], evaluated the sensitivity and PPV of ICD-9 410 over the period 1987–2000. They reported that while overall, these statistics remained relatively stable, amongst teaching hospitals they declined significantly (with sensitivity declining from 74% to 59%, and PPV from 80% to 71%). In contrast, in a study conducted at a university hospital in the Netherlands, both sensitivity and PPV were higher in the later period (years 1996–2003) than the earlier period 1987–1995 (with sensitivity increasing from 82% to 85%, and PPV from 94% to 99%) [62].

In addition to being more sensitive, cardiac troponin is also a more specific indicator of MI. Although few studies in this review reported specificity values directly, this statistic can be analysed by way of PPV. Specificity is equal to 1 - the number of false positives, so will increase as the number of false-positive cases decreases. PPV is the proportion of true-positives amongst all true-positive and false-positive cases, so will also increase as the number of false-positive cases decreases. The fact that the PPV's for hospitalization data generally increased over time provides support for an increase in the specificity of MI diagnoses as well.

When comparing the performance of the newer diagnostic criteria to the MONICA, the contribution of other secular changes must be considered. One factor is the use of different revisions of the ICD coding system in different time periods. Mahonen *et al* [35] found that the sensitivity of ICD 410 was generally lower during the period 1987–1990 (ICD-9) than 1983–1986 (ICD-8), even though the same diagnostic critera (FINMONICA, a Finnish adaptation of the MONICA criteria) were used throught the study period. In contrast, those authors found that the PPV's in the ICD-9 period were generally higher than in the ICD-8 period. However, the impact that cardiac troponin testing has on the validity of MI diagnoses is difficult to ignore. For example, Pajunen *et al* [43] reported higher sensitivity during the ICD-10 period (1998–2002) than the ICD-9 period (1988–1997), but the authors attribute this difference to the use of cardiac troponin testing during the ICD-10 period. We believe the introduction of cardiac troponin testing and its increasing use over time may be mainly responsible for the improvements we observed in the PPV of MI codes over time.

When examining only studies that used the MONICA criteria, we observed that the PPV's were usually higher in studies stemming from the original MONICA project compared to those just applying the MONICA criteria in other samples. This was especially apparent amongst the European studies from the MONICA project. One explanation for this may be some cross-referencing between the hospital databases and MONICA registries. It is acknowledged in these studies [21], [35] how the MONICA project itself may have influenced local coding practices. For example, some of the same physicians that were involved with the MONICA study were also treating patients hospitalized for coronary events in local centres. However the potential influence these factors may have had in Europe, they did not appear to carry over in Australia and New Zealand, where the PPV's in studies using the MONICA registries were much lower.

We observed that the accuracy of MI as a cause of death on death certificates was lower in comparison to hospitalization data. Death certificate diagnoses of MI may be less accurate because less information is available on these cases from which to determine a precise cause of death. Specifically, many deaths are not attended to by medical personnel, resulting in a lack of comprehensive documentation [39]. In support of this, Lowel *et al* [41] found that the PPV's were lower for cases who spent less time in hospital, and had less clinical data and test results (including electrocardiograms and enzyme levels) available, which could otherwise aide in establishing a more accurate cause of death [41].

Our review showed that the accuracy of hospitalization data for identifying MI cases is much higher than data from death certificates; consequently, we recommend that, when available, researchers attempt to confirm the cause of death by matching vital statistics death records for MI with administrative hospitalization data. At the very least, the limitations of vital statistics data should be acknowledged by these authors.

Many of the findings presented in this paper are based on PPV, which was the most frequently-reported statistic amongst the studies included in this review. PPV is relatively easy for researchers to assess since they only need to evaluate cases who initially test positive for the condition (here being MI). However, a caveat of both PPV and NPV are their dependence on the prevalence of the condition in the study population [63]. The PPV will be lower for a rare condition than for a common condition. For example, amongst all testing positive in a rare condition (those in the denominator), few are likely to be true-positives (and appear in the numerator). In this review, we expected the PPV's to be lower amongst the community-based studies than the clinic-based studies or those with otherwise more selected populations, and this was apparent in several studies. For instance, the PPV in a study of patients admitted to coronary care units was 89% [48] and in two studies that were restricted to individuals aged 65 years and older (amongst whom MI is more common) the PPV's were 95% [47] and 98% [45]. In contrast, in another study which had a younger source population (aged between 25 and 64 years), the PPV was much lower (only 67%) [37]. Consequently, differences in the expected prevalence of MI in the different source populations may have contributed to variation in the PPV's reported by the different studies in this review.

A significant research gap was identified in the course of this review, being a lack of studies reporting on the validity of codes from the ICD-10. This system has been in widespread use in Europe and Australia for at least a decade, but ICD-10 codes were evaluated in just three studies included in this review, and only two of these [42], [43] reported on the validity of ICD-10 codes separately from ICD-9 codes. One of these studies reported that the PPV for ICD-10 I21-22 was good, especially in tertiary care hospitals (PPV = 93%) [42], and findings from the other suggest that ICD-10 I21-22 is more sensitive for MI than the equivalent ICD-9 code, 410 [43]. With ICD-10 codes now a key component of health research, assessments of the validity of ICD-9 codes are quickly losing their relevance, and clearly, more investigations into the accuracy of ICD-10 codes are needed to support ongoing research endeavours.

Our systematic review has some limitations. We could not consider articles whose full-text was not available in English, and this may have introduced a language bias. We were unable to include articles that did not report or reference the diagnostic algorithms being validated, or those that were published after the conclusion of our search period (February 2011). As well, although our MEDLINE and EMBASE searches were conducted by an experienced librarian, some relevant studies may have been missed since administrative databases are not well catalogued in these indexes (e.g. no MeSH term pertaining to “administrative database”). Most of the articles included in this review were located through database searches. In these, we searched for articles that were indexed under terms relating to Administrative Data, Validation, and Cardiovascular Disease. However, in our subsequent handsearch we located several relevant articles that were not indexed under these Administrative Data or Validation categories. Thus, while our handsearches were extensive, it is possible that we still missed some relevant articles if they were not indexed in the databases with a term relating to validation or administrative data, or were published in a journal not indexed in the MEDLINE or EMBASE databases.

In summary we conclude that, based on the evidence, hospitalization data can be used to identify MI as a covariate or outcome, but the accuracy of MI as a cause-of-death in vital statistics data is limited. Authors using vital statistics data to identify MI deaths are encouraged to compare such data with hospitalization data to confirm the cause of death or use sensitivity analyses excluding cases from this source. While most administrative databases are not established for research purposes, they are increasingly being used to study long-term patient outcomes and disease burden. Therefore, in order to maximize the sensitivity of these databases, physicians and hospital coders should be encouraged to record all significant complications and comorbidities. In the meantime, authors using administrative data to identify MI deaths should acknowledge the limitations of this data source. Finally, with ICD-10 coding now commonplace, more assessments of the validity of ICD-10 codes for MI are needed to ensure the quality of future research. We believe our findings will help to increase the rigour of population-based epidemiological and outcomes research and thus potentially improve health surveillance, resource allocation and patient care.

## Supporting Information

Table S1 Item-by-item quadas breakdown for each study.
(DOCX)

Text S1 MEDLINE search strategy.
(DOCX)

Text S2 EMBASE search strategy.
(DOCX)

Text S3 Data collection form.
(DOC)

Checklist S1 PRISMA Checklist.
(DOC)
