# Supplementary material for: Validity of Myocardial Infarction Diagnoses in Administrative Databases: A Systematic Review
Source: PLoS One. 2014 Mar 28;9(3):e92286. doi: 10.1371/journal.pone.0092286 (PMC3969323; doi:10.1371/journal.pone.0092286)
Supplement: Table S1 — Item-by-item quadas breakdown for each study. (DOCX) [file pone.0092286.s001.docx]

**Table S1: Item-by-Item QUADAS Breakdown for Each Study**

| **QUADAS Question no.** | **Ainla**  [42] | **Austin** [48] | **Barchielli** [49] | **Beaglehole** [37] | **Boyle** [20] | **De Henauw** [39] | **Hammar** [64] | **Heckbert** [65] | **Jackson** [38] | **Kennedy** [40] | **Kiyota**  [47] | **Levy**  [45] | **Lindblad**  [66] | **Lowel** [41] | **Mahonen (1997)** [35] |
| --- | --- | --- | --- | --- | --- | --- | --- | --- | --- | --- | --- | --- | --- | --- | --- |
| **1** | Y | Y | Y | Y | Y | Y | Y | N | Y | Y | N | N | Y | Y | Y |
| **2** | Y | Y | Y | Y | Y | Y | Y | Y | Y | Y | Y | Y | Y | Y | Y |
| **3** | Y | Y | Y | Y | Y | Y | Y | Y | Y | Y | Y | N | Y | Y | Y |
| **4** | NA | NA | NA | NA | NA | NA | NA | NA | NA | NA | Y | NA | NA | NA | NA |
| **5** | Y | Y | Y | Y | Y | Y | Y | Y | Y | Y | Y | Y | Y | Y | Y |
| **6** | NA | Y | Y | Y | Y | Y | Y | Y | Y | Y | Y | Y | Y | Y | Y |
| **7** | N | Y | N | Y | Y | Y | Y | Y | Y | Y | N | N | N | N | Y |
| **8** | Y | Y | Y | Y | Y | Y | Y | Y | Y | Y | Y | Y | Y | Y | Y |
| **9** | N | Y | Y | Y | U | Y | Y | N | U | Y | Y | Y | Y | Y | Y |
| **10** | Y | Y | Y | Y | Y | Y | Y | Y | Y | Y | Y | Y | Y | Y | Y |
| **11** | NA | Y | U | U | Y | Y | U | U | U | Y | U | NA | U | U | Y |
| **12** | U | Y | Y | Y | Y | Y | Y | Y | U | Y | Y | Y | Y | Y | Y |
| **13** | NA | Y | NA | NA | Y | Y | Y | U | Y | NA | Y | NA | Y | Y | NA |
| **14** | Y | NA | NA | NA | NA | NA | NA | NA | NA | NA | NA | NA | NA | NA | NA |
| **Overall Quality Assessment** | High | High | High | High | High | High | High | High | High | High | Medium | Medium | High | High | High |

Y=Yes; N=No; U=Unclear; NA=Not Applicable

| **QUADAS Question no.** | **Mahonen (1999)** [34] | **McCarthy** [44] | **Merry** [62] | **Nova Scotia (1989)** [51] | **Nova Scotia (1992)** [50] | **Pajunen** [43] | **Palomaki** [21] | **Petersen** [67] | **Pladevall** [22] | **Rapola**  [46] | **Rawson**  [68] | **Rosamond** [36] | **van Walraven** [69] | **Varas-Lorenzo** [70] | **Wahl** [71] |
| --- | --- | --- | --- | --- | --- | --- | --- | --- | --- | --- | --- | --- | --- | --- | --- |
| **1** | Y | N | Y | Y | Y | Y | Y | N | Y | N | Y | Y | Y | Y | Y |
| **2** | Y | Y | Y | Y | Y | Y | Y | Y | Y | Y | Y | Y | Y | Y | Y |
| **3** | Y | U | Y | Y | Y | Y | Y | Y | Y | Y | U | Y | Y | Y | Y |
| **4** | NA | NA | NA | NA | NA | NA | NA | NA | NA | NA | NA | NA | NA | NA | NA |
| **5** | Y | Y | Y | Y | Y | Y | Y | Y | Y | Y | Y | Y | Y | Y | Y |
| **6** | Y | Y | Y | NA | Y | Y | Y | Y | Y | Y | Y | Y | Y | Y | Y |
| **7** | N | Y | Y | N | Y | N | N | N | Y | N | N | Y | N | N | N |
| **8** | Y | Y | Y | Y | Y | Y | Y | Y | Y | Y | Y | Y | Y | Y | Y |
| **9** | Y | N | Y | U | U | NA | Y | Y | U | U | N | Y | Y | Y | N |
| **10** | Y | Y | Y | Y | Y | Y | Y | Y | Y | Y | Y | Y | Y | Y | Y |
| **11** | Y | N | Y | NA | U | Y | U | NA | U | U | Y | U | NA | U | Y |
| **12** | Y | Y | Y | Y | Y | Y | Y | Y | Y | Y | Y | Y | Y | Y | Y |
| **13** | Y | Y | U | Y | NA | NA | NA | NA | NA | Y | NA | NA | Y | Y | NA |
| **14** | NA | NA | NA | NA | NA | NA | NA | NA | NA | NA | NA | NA | NA | NA | NA |
| Overall Quality Assessment | High | Medium | High | High | High | High | High | High | High | Medium | High | High | High | High | High |

Y=Yes; N=No; U=Unclear; NA=Not Applicable

*QUADAS questions are displayed in Text S3
