# Supplementary material for: Validity of Myocardial Infarction Diagnoses in Administrative Databases: A Systematic Review
Source: PLoS One. 2014 Mar 28;9(3):e92286. doi: 10.1371/journal.pone.0092286 (PMC3969323; doi:10.1371/journal.pone.0092286)
Supplement: Text S1 — MEDLINE search strategy. (DOCX) [file pone.0092286.s002.docx]

**Text S1: MEDLINE search strategy (inception to November 2010)**

Database: Ovid MEDLINE(R) 1950 to Present with Daily Update

Search Strategy:

--------------------------------------------------------------------------------

1 administrative data.ti,ab. (2259)

2 administrative database:.ti,ab. (1250)

3 Databases, Factual/ (30835)

4 factual database/ (30835)

5 Databases as Topic/ (7263)

6 database/ (0)

7 Medical Record Linkage/ (2778)

8 administrative databank:.ti,ab. (2)

9 factual database:.ti,ab. (17)

10 factual databank:.ti,ab. (2)

11 factual data.ti,ab. (46)

12 exp medical records/ (69226)

13 exp medical record/ (69226)

14 exp medical records systems, computerized/ (18265)

15 (medical record or health record or medical records or health records).ti,ab. (43624)

16 medical transcription:.ti,ab. (71)

17 exp Registries/ (42218)

18 registry/ (39941)

19 (registry or registries).ti,ab. (39648)

20 (utilization data: or utilisation data: or claims data: or managed care data: or physician billing data: or hospitalization data: or linked data:).ti,ab. (5216)

21 (administrative healthcare data: or administrative health care data: or administrative health data: or administrative health data:).ti,ab. (136)

22 (medical records based index or claims based index).ti,ab. (5)

23 (register and (link or links or linked or linkage or linking)).ti,ab. (1775)

24 or/1-23 [ADMINISTRATIVE DATA (BROAD)] (202114)

25 Validation Studies/ (47936)

26 validation study/ (0)

27 Validation Studies as Topic/ (588)

28 Validation Studies.pt. (47936)

29 (validat: or validity).ti,ab. (219858)

30 or/25-29 [VALIDATION STUDIES] (240669)

31 or/1-2,8,21 [ADMINISTRATIVE DATA (NARROWEST)] (3485)

32 Coronary Artery Disease/ (26709)

33 coronary artery disease/ (26709)

34 coronary artery disease:.ti,ab. (47741)

35 Myocardial Infarction/ (126134)

36 acute heart infarction/ (0)

37 acute myocardial infarction:.ti,ab. (39458)

38 exp Heart Failure/ (71069)

39 exp congestive heart failure/ (71069)

40 congestive heart failure.ti,ab. (27488)

41 exp Stroke/ (61840)

42 stroke/ (38759)

43 ((stroke or strokes) and (brain or cerebral or cerebrovascular)).ti,ab. (32188)

44 Brain Ischemia/ (29595)

45 brain ischemia/ (29595)

46 ((brain or cerebral or cerebrovascular) adj2 (vascular accident: or apoplex: or infarction: or ischemi:)).ti,ab. (34223)

47 (cerebrovascular event or cerebrovascular events).ti,ab. (1915)

48 or/32-47 [CARDIOVASCULAR (SPECIFIC)] (362328)

49 24 and 30 and 48 [ADMINISTRATIVE DATA (BROAD) + VALIDATION STUDIES + CARDIOVASCULAR (SPECIFIC)] (548) **SAVED EN 1-548 (519 UNIQUE)**

50 31 and 48 [ADMINISTRATIVE DATA (NARROWEST) + CARDIOVASCULAR (SPECIFIC)] (388)

51 49 or 50 (880) **(832 UNIQUE)**

52 50 not 49 (332) **SAVED EN 549-880 (313 UNIQUE)**
