# Supplementary material for: Validity of Myocardial Infarction Diagnoses in Administrative Databases: A Systematic Review
Source: PLoS One. 2014 Mar 28;9(3):e92286. doi: 10.1371/journal.pone.0092286 (PMC3969323; doi:10.1371/journal.pone.0092286)
Supplement: Text S2 — EMBASE search strategy. (DOCX) [file pone.0092286.s003.docx]

**Text S2: EMBASE search strategy (inception to November 2010)**

Database: EMBASE <1980 to 2010 Week 44>

--------------------------------------------------------------------------------

1 administrative data.ti,ab. (4916)

2 administrative database:.ti,ab. (2827)

3 Databases, Factual/ (41390) MESH

4 factual database/ (41390) EMBASE

5 Databases as Topic/ (78922)

6 database/ (71659)

7 Medical Record Linkage/ (92531)

8 administrative databank:.ti,ab. (4)

9 factual database:.ti,ab. (41)

10 factual databank:.ti,ab. (6)

11 factual data.ti,ab. (110)

12 exp medical records/ (158979)

13 exp medical record/ (158979)

14 exp medical records systems, computerized/ (108018)

15 (medical record or health record or medical records or health records).ti,ab. (96112)

16 medical transcription:.ti,ab. (150)

17 exp Registries/ (73539)

18 registry/ (71261)

19 (registry or registries).ti,ab. (88075)

20 (utilization data: or utilisation data: or claims data: or managed care data: or physician billing data: or hospitalization data: or linked data:).ti,ab. (11860)

21 (administrative healthcare data: or administrative health care data: or administrative health data: or administrative health data:).ti,ab. (295)

22 (medical records based index or claims based index).ti,ab. (10)

23 (register and (link or links or linked or linkage or linking)).ti,ab. (3836)

24 or/1-23 [ADMINISTRATIVE DATA (BROAD)] (465796)

25 Validation Studies/ (72021)

26 validation study/ (24075)

27 Validation Studies as Topic/ (24663)

28 Validation Studies.pt. (47946)

29 (validat: or validity).ti,ab. (503193)

30 or/25-29 [VALIDATION STUDIES] (531950)

31 or/1-2,8,21 [ADMINISTRATIVE DATA (NARROWEST)] (7677)

32 Coronary Artery Disease/ (144013)

33 coronary artery disease/ (144013)

34 coronary artery disease:.ti,ab. (106198)

35 Myocardial Infarction/ (282356)

36 acute heart infarction/ (33814)

37 acute myocardial infarction:.ti,ab. (86390)

38 exp Heart Failure/ (264166)

39 exp congestive heart failure/ (132064)

40 congestive heart failure.ti,ab. (59449)

41 exp Stroke/ (150366)

42 stroke/ (127285)

43 ((stroke or strokes) and (brain or cerebral or cerebrovascular)).ti,ab. (73580)

44 Brain Ischemia/ (76645)

45 brain ischemia/ (76645)

46 ((brain or cerebral or cerebrovascular) adj2 (vascular accident: or apoplex: or infarction: or ischemi:)).ti,ab. (77383)

47 (cerebrovascular event or cerebrovascular events).ti,ab. (4479)

48 or/32-47 [CARDIOVASCULAR (SPECIFIC)] (968745)

49 24 and 30 and 48 [ADMINISTRATIVE DATA (BROAD) + VALIDATION STUDIES + CARDIOVASCULAR (SPECIFIC)] (1364)

50 31 and 48 [ADMINISTRATIVE DATA (NARROWEST) + CARDIOVASCULAR (SPECIFIC)] (939)

51 49 or 50 (2173)

52 50 not 49 (809)

53 remove duplicates from 49 (968)

54 from 53 keep 1-443 (443) **SAVED EN 881-1323 (438 UNIQUE)**

55 remove duplicates from 52 (481)

56 from 55 keep 1-162 (162) **SAVED EN 1324-1485 (157 UNIQUE)**
