# Supplementary material for: Validity of Myocardial Infarction Diagnoses in Administrative Databases: A Systematic Review
Source: PLoS One. 2014 Mar 28;9(3):e92286. doi: 10.1371/journal.pone.0092286 (PMC3969323; doi:10.1371/journal.pone.0092286)
Supplement: Text S3 — Data collection form. (DOC) [file pone.0092286.s004.doc]

**Data Collection Instrument for comorbidities using administrative data (Group 3)**

Study Citation:

| **General information** | | | |
| --- | --- | --- | --- |
| **Title** |  | | |
| **Authors** |  | | |
| **Country** | Canada? Yes ⬜ No ⬜ If no, specify: _____________________ | | |
| **Journal** |  | | |
| **Year** |  | Issue | Page |
| **Funding support** |  | | |

**Please tick as many as appropriate and write when appropriate**

| **Condition validated** | ⬜ Cardiovascular disease (combine outcome)  ⬜ AMI,  ⬜ Stroke  ⬜ Transient Ischemic Attack  ⬜ CHF  ⬜ Diabetes  ⬜ Deep venous thrombosis  ⬜ Renal Failure  ⬜ Osteoporosis  ⬜ Osteoporotic fractures  ⬜ Cancer (specify):  _______________________  _______________________  _______________________  ⬜ Infections (specify):  _______________________  _______________________  _______________________  ⬜ Other (specify)  _______________________  _______________________  _______________________ | | | | | | | |
| --- | --- | --- | --- | --- | --- | --- | --- | --- |
| Describe study population for administrative data source (e.g. RA population,general population with specific diagnosis, or hospital patients with specific diagnoses):  ______________________________________________________________________________________________________________________________________________________________________________________________________________________________  Was the condition of interest: ⬜ their primary disease ⬜ or a comorbidity | | | | | | | | |
| Type of administrative data for identifying comorbid condition | | **Please tick all that apply and write when appropriate**   - **Inpatient records**: hospital records   - Hospital separation files (admin data from hospitalization)   - Electronic medical records from hospitalization - **Outpatient records**:   - Admin data on physician services (e.g. billing, procedures codes etc)   - Admin data on investigations   - Admin data on medications   - Electronic medical records from physician visits - **Vital Statistics** - **Cancer registry** - **Other (specify)**   _________________________________________  _________________________________________  _________________________________________ | | | | | | |
| **Case definition of the comorbidity** | | Please **write** the algorithm used in this article to define the comorbidity. | | | | | | |
| **Case definition includes: (circle when appropriate)** | | | | **Yes** | **No** | **Not stated** | **Not applicable** | |
| 1. Use of diagnostic codes | | | |  |  |  |  | |
| 1. Diagnostic code evaluated was only primary or most responsible Dx. | | | |  |  |  |  | |
| 1. Exclusion of specific diagnoses | | | |  |  |  |  | |
| 1. Use of medication | | | |  |  |  |  | |
| 1. Use of procedures/interventions/ confirmatory test | | | |  |  |  |  | |
| 1. A specific time frame | | | |  |  |  |  | |
| Performed a Sensitivity Analysis of Case Definition  (or confirms the diagnosis in a subset of patients) | | | | | | Yes | | No |
| **VALIDATION METHOD:** | | | | | | | | |
| **1) Source of data for validation**  **2) Diagnosis for gold standard is based on:** | | | - Review of Medical Record - Asking MD to confirm diagnosis - Self-report by patient - Evaluation of patients by investigators - Other: _____________________   ⬜ MD impression   - Confirmatory tests / investigation - Meets diagnostic criteria - Impression of reviewer - Other: _____________________ | | | | | |
| **Statistic used and result** | | | (If 95% CI available please write them)  Sensitivity __________________  Specificity __________________  Positive predictive value __________________  Negative predictive value __________________  Kappa _______  Other (specify): ___________________  ___________________ | | | | | |

**QUALITY ASSESSMENT**

| Please consider “the test” as the diagnostic algorithm used in the paper. | | | | | |
| --- | --- | --- | --- | --- | --- |
| **The QUADAS tool** | | | | | |
| **Item** |  | **Yes (1)** | **No (0)** | **Unclear (0)** | **N/A (0)** |
| ------------------------------------------------------------------------------------------------------------------------------------------------------ | | | | | |
| 1. | Was the spectrum of patients representative of the patients who will receive the comorbidity diagnosis in practice? | ( ) | ( ) | ( ) | ( ) |
| 2. | Were selection criteria clearly described? | ( ) | ( ) | ( ) | ( ) |
| 3. | Is the reference standard likely to correctly classify the target condition? | ( ) | ( ) | ( ) | ( ) |
| 4. | Is the time period between reference standard and index test short enough to be reasonably sure that the target condition did not change between the two tests? | ( ) | ( ) | ( ) | ( ) |
| 5. | Did the whole sample or a random selection of the sample, receive verification using a reference standard of diagnosis? | ( ) | ( ) | ( ) | ( ) |
| 6. | Did patients receive the same reference standard regardless of the index test result? | ( ) | ( ) | ( ) | ( ) |
| 7. | Was the reference standard independent of the index test (i.e. the index test did not form part of the reference standard)? | ( ) | ( ) | ( ) | ( ) |
| 8. | Was the execution of the index test described in sufficient detail to permit replication of the test? | ( ) | ( ) | ( ) | ( ) |
| 9. | Was the execution of the reference standard described in sufficient detail to permit its replication? | ( ) | ( ) | ( ) | ( ) |
| 10. | Were the index test results interpreted without knowledge of the results of the reference standard? | ( ) | ( ) | ( ) | ( ) |
| 11. | Were the reference standard results interpreted without knowledge of the results of the index test? | ( ) | ( ) | ( ) | ( ) |
| 12. | Were the same clinical data available when test results were interpreted as would be available when the test is used in practice? | ( ) | ( ) | ( ) | ( ) |
| 13. | Were uninterpretable/ intermediate test results reported? | ( ) | ( ) | ( ) | ( ) |
| 14. | Were withdrawals from the study explained? | ( ) | ( ) | ( ) | ( ) |
|  | | | | | |
|  | **Total Score**  **Sum of applicable items: _______ Number of N/A items: ______** | | | | |
|  | **Please select your opinion of the overall quality of this study** | Poor Excellent  (1) (2) (3) (4) (5) | | | |
| Comments | | | | | |
